# Single-Cell RNA Sequencing Reveals T-Cell Metabolic Activation and SGPL1 as a Candidate Predictor of PD-1 Resistance in Urothelial Carcinoma

**DOI:** 10.3390/ph19081284

**Published:** 2026-08-14

**Authors:** Zheng Chao, Yuchen Mei, Hao Peng, Guowen Li, Xiaodong Hao, Yuchuan Liu, Guanglu Lyu, Zhihua Wang, Le Li

**Affiliations:** 1 Department of Urology, Hubei Provincial Clinical Medical Research Center for Minimally Invasive Treatment of Urology, Tongji Hospital, Tongji Medical College and State Key Laboratory for Diagnosis and Treatment of Severe Zoonotic Infectious Diseases, Huazhong University of Science and Technology, Wuhan 430030, China; 2 Department of Urology, South China Hospital, Shenzhen University, Shenzhen 518111, China

**Keywords:** urothelial carcinoma, PD-1 resistance, single-cell RNA sequencing, metabolic reprogramming, SGPL1, macrophage polarization

## Abstract

**Background/Objectives**: Immune checkpoint blockade targeting the PD-1/PD-L1 axis is a standard treatment for urothelial carcinoma (UC), but 20–30% of patients develop resistance. The underlying mechanisms, particularly regarding tumor microenvironment metabolic reprogramming, remain unclear. **Methods**: We performed single-cell RNA sequencing on pre-treatment tumors from eight pembrolizumab-treated UC patients (three responders, five non-responders). Bioinformatic analyses included cell clustering, pathway enrichment, and cell–cell communication, validated using public datasets. Functional experiments involved SGPL1 knockdown in MB49 cells and a syngeneic PD-1-resistant murine model (MB49-R5). **Results**: Responders exhibited a higher proportion of infiltrating T-cells. Unexpectedly, CD4^+^ subsets, rather than CD8^+^ cells, were numerically enriched in responders. However, all T-cell subsets in responders demonstrated enhanced oxidative phosphorylation-dominant metabolic activity. Enhanced macrophage–T-cell crosstalk was observed, with macrophages exhibiting M1-like polarization driven by CD4^+^ T-cell-derived IFN-γ and CD40L signaling. In contrast, non-responders displayed dominant tumor-derived MIF–CD74 signaling and M2 polarization. Integration of public datasets identified SGPL1 as a key metabolic regulator associated with poor prognosis and reduced immune activation. Functional experiments demonstrated that SGPL1 knockdown inhibited tumor proliferation and restored anti-PD-1 sensitivity, accompanied by increased T-cell infiltration. **Conclusions**: T-cell metabolic activation drives anti-PD-1 responses in UC, and CD4^+^ T-cell-mediated M1 polarization and tumor-intrinsic SGPL1 crucially shape therapeutic outcomes, highlighting SGPL1 as a candidate metabolic target to overcome PD-1 resistance.

## 1. Introduction

Urothelial carcinoma (UC) is the most common malignancy of the urinary tract, arising from urothelial epithelial cells and predominantly affecting the bladder, renal pelvis, ureter, and urethra [1]. Bladder cancer accounts for more than 90% of UC cases and represents a major global health burden [2].

Immune checkpoint blockade targeting the PD-1/PD-L1 axis has significantly improved outcomes in UC, and is now widely used as a frontline therapeutic strategy [3]. However, only a subset of patients derives durable clinical benefit, with approximately 20–30% exhibiting primary or acquired resistance [4]. Tumor immune evasion is a major contributor to treatment failure and is closely associated with dynamic alterations in the tumor immune microenvironment (TME) [5].

CD8^+^ T-cells constitute the principal target population of PD-1–directed therapy, particularly stem-like progenitor exhausted T-cells (Tpex), which retain proliferative capacity upon immune reinvigoration [6]. CD4^+^ T-cells have traditionally been viewed as helper cells orchestrating humoral immunity; however, accumulating evidence demonstrates their capacity to mediate antitumor immunity either directly or indirectly in coordination with CD8^+^ T-cells [7,8]. Macrophages, a highly heterogeneous population defined by diverse functional states, exhibit remarkable plasticity between tumor-promoting and tumor-suppressive phenotypes [9]. Although refined transcriptional programs are increasingly used to define macrophage states, the classical M1/M2 paradigm remains a useful framework for distinguishing inflammatory and immunosuppressive polarization [10,11].

In the context of PD-1 resistance, multiple signaling pathways have been implicated in driving M1-to-M2 polarization, among which metabolic crosstalk within the TME represents a critical mechanism of intercellular communication [12]. Dysfunctional CD8^+^ T-cells typically exhibit impaired mitochondrial respiration and reduced bioenergetic capacity, rendering them refractory to PD-1 reinvigoration [13]. In contrast, effector T-cells depend on oxidative phosphorylation (OXPHOS) to sustain antitumor activity [14]. Tumor cells, however, actively subvert immune cell metabolism through nutrient competition and the accumulation of immunosuppressive metabolites, thereby crippling antitumor immune responses [13,15].

Notably, dysregulated sphingolipid metabolism promotes excessive consumption of sphingosine-1-phosphate (S1P) in the TME, thereby sustaining tumor growth while impairing immune cell function [16]. S1P receptor-1 (S1P1), the most widely expressed S1P receptor, promotes regulatory T-cell expansion and correlates with poor prognosis in bladder cancer [17]. S1P homeostasis is tightly controlled by sphingosine-1-phosphate lyase (SGPL1), the only enzyme responsible for irreversible S1P degradation [18,19]. SGPL1 has been implicated in cancer progression by redirecting sphingolipid metabolites toward glycerophospholipid biosynthesis [20]. However, whether SGPL1-mediated metabolic reprogramming contributes to PD-1 resistance in UC remains unclear.

In this study, we established an in-house cohort of UC patients treated with PD-1 blockade and performed single-cell RNA sequencing on tumor samples from eight individuals. We found that responders exhibited markedly enhanced crosstalk between CD4^+^ T-cells and macrophages, which correlated positively with M1 macrophage polarization. Concurrently, CD8^+^ T-cells in responders displayed robust metabolic activation, and their antitumor efficacy was strongly associated with CD4^+^ T-cell functionality. We further identified sphingosine-1-phosphate lyase 1 (SGPL1) as a critical metabolic therapeutic target. SGPL1 promotes S1P degradation, thereby fostering tumor proliferation and invasion while suppressing antitumor immunity. Importantly, targeted knockdown of SGPL1 significantly sensitized tumors to PD-1 blockade, offering a promising combinatorial strategy to overcome resistance.

## 2. Results

### 2.1. Metabolic Activation Distinguishes T-Cell Responses to Anti-PD-1 Therapy in Urothelial Carcinoma

**Figure 1:**
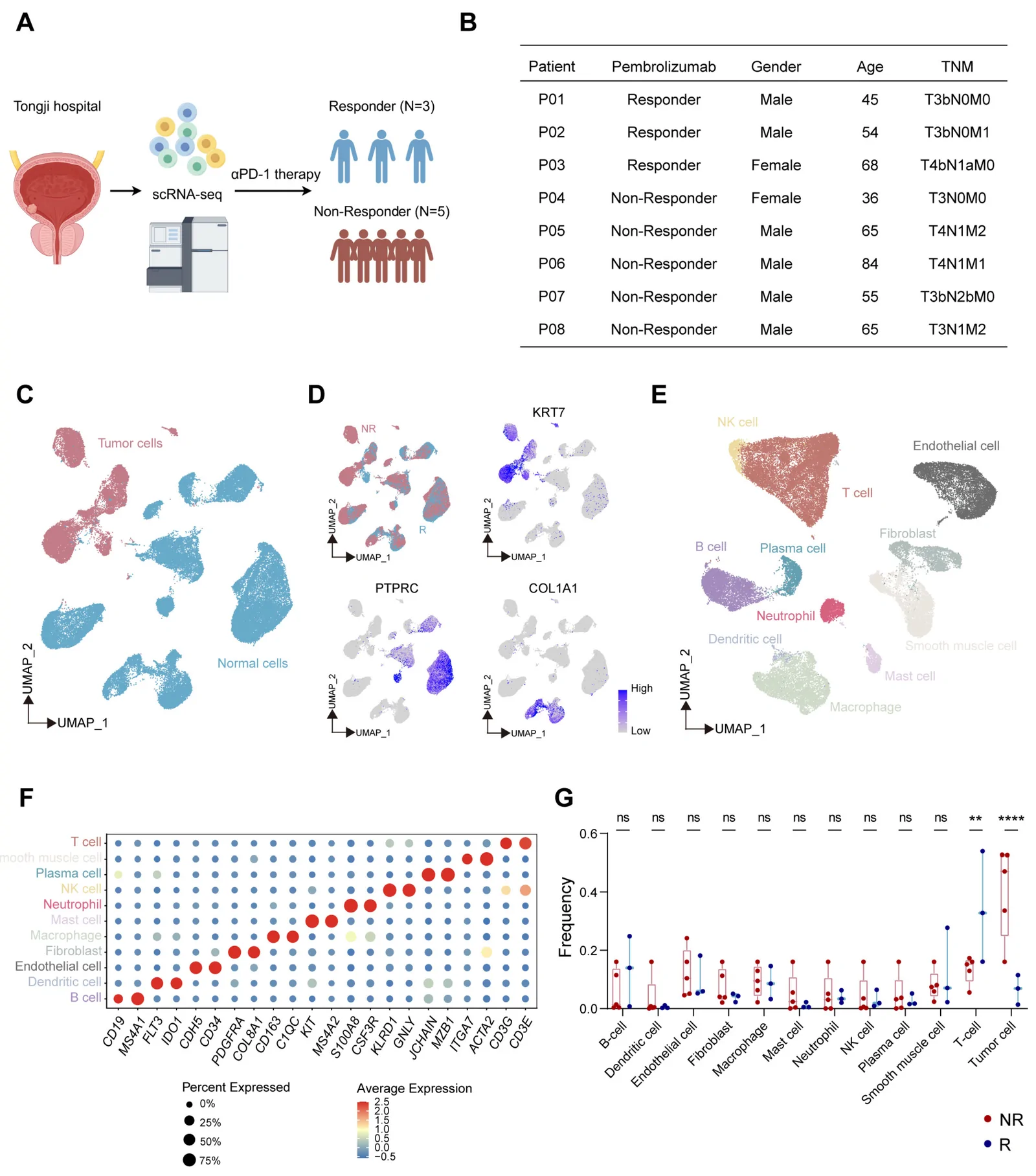
**Single-cell landscape of the tumor microenvironment in urothelial carcinoma patients treated with anti-PD-1 therapy**. (**A**) Schematic illustration of the study design. Pre-treatment tumor samples were collected from bladder cancer patients and subjected to single-cell RNA sequencing (scRNA-seq). Patients were stratified into responder (R) and non-responder (NR) groups based on clinical response to PD-1 blockade. (**B**) Baseline clinical characteristics of the enrolled patients. (**C**) Uniform Manifold Approximation and Projection (UMAP) visualization of all cells colored by response identity. (**D**) UMAP plots showing the distribution of NR and R samples (left), and expression of lineage markers KRT7 (urothelial carcinoma cells), PTPRC (CD45, immune cells), and COL1A1 (fibroblasts). (**E**) UMAP visualization of normal cells with annotated cell types. (**F**) Dot plots showing the expression of canonical markers for each immune cell population. (**G**) Proportional analysis of major cell types. T-cells were significantly increased in the R group, whereas tumor cells were decreased. ns: Not Significant, **: *p* < 0.01, ****: *p* < 0.0001.

We performed single-cell RNA sequencing on pre-treatment tumor specimens from eight patients with urothelial carcinoma treated with pembrolizumab (three responders and five non-responders; primary and metastatic lesions) (Figure 1A,B) [21]. Using canonical lineage markers (KRT7, PTPRC, COL1A1), we partitioned cells into epithelial, immune, and stromal compartments, followed by high-resolution immune subclustering (Figure 1C–F). T-cells constituted a significantly larger fraction of the immune infiltrate in responders (Figure 1G).

**Figure 2:**
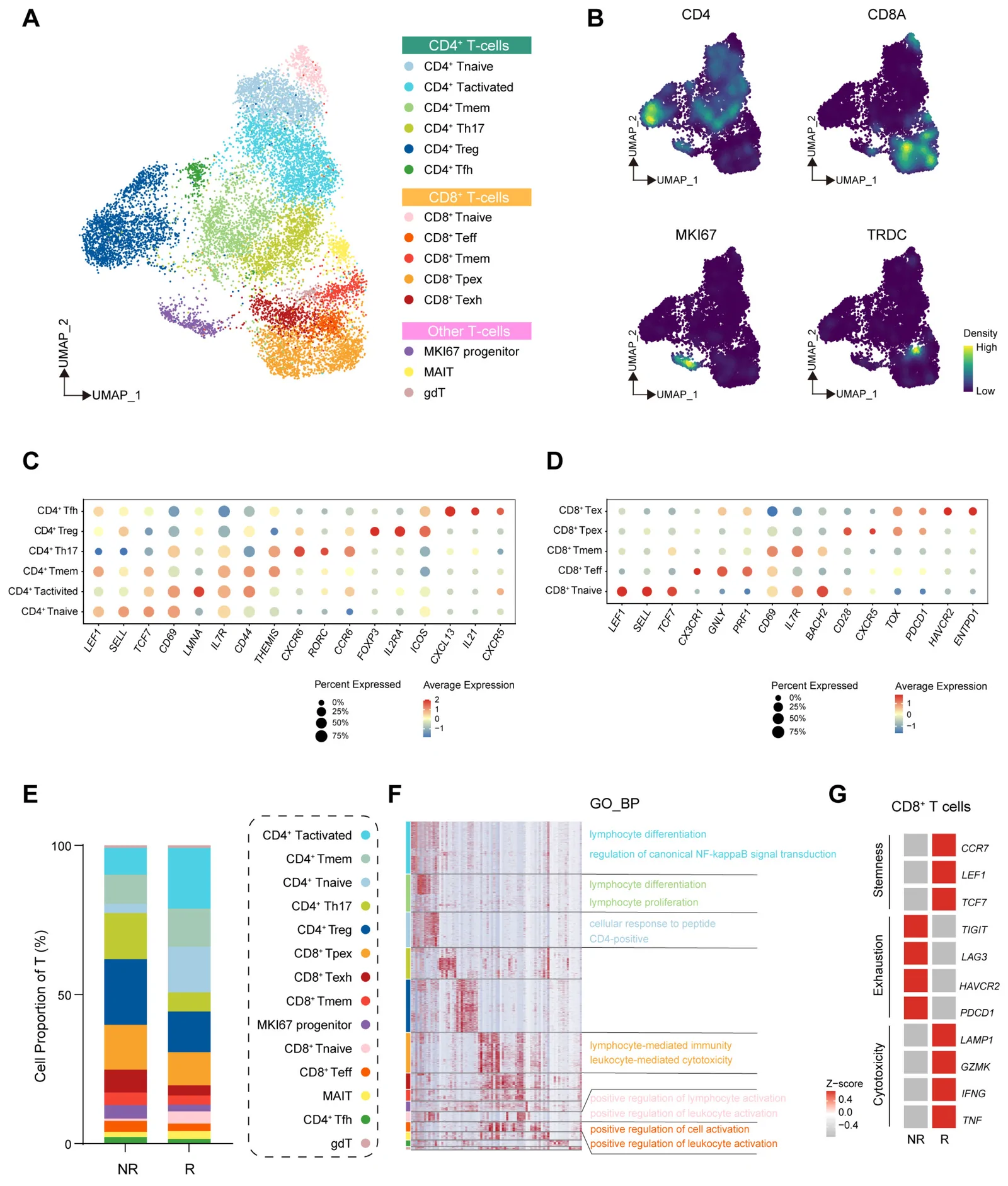
**T-cell subset composition and metabolic activation in responders and non-responders.** (**A**) UMAP visualization of T-cell clusters after re-clustering. (**B**) UMAP plots showing the distribution of CD4^+^ T-cells, CD8^+^ T-cells, MKI67^+^ proliferating T-cells, and γδ T-cells. (**C**) UMAP visualization and marker expression of CD4^+^ T-cell subpopulations. (**D**) UMAP visualization and marker expression of CD8^+^ T-cell subpopulations. (**E**) Quantification of T-cell subset proportions. CD4^+^ activated T-cells were enriched in responders, whereas Th17 and Treg cells were reduced. (**F**) Heatmap of signature genes for each T-cell subset and Gene Ontology Biological Process (GO_BP) enrichment analysis of marker genes for CD4^+^ activated, CD4^+^ memory, CD4^+^ naïve, CD8^+^ effector, and CD8^+^ progenitor exhausted (pex) cells. (**G**) Stemness, cytotoxicity, and exhaustion scores for CD8^+^ T-cells. The R group exhibited higher stemness and cytotoxicity, with lower exhaustion levels.

Upon further stratification into CD4^+^ subsets (activated, memory, naïve), CD8^+^ subsets (effector, progenitor exhausted), and minor populations (γδ T and MAIT-cells) (Figure 2A–D), we unexpectedly found that responders were enriched in CD4^+^ subsets rather than CD8^+^ effector or progenitor exhausted cells (Figure 2E,F). Despite that, CD8^+^ T-cells from responders exhibited a stemness- and cytotoxicity-biased phenotype over exhaustion (Figure 2G). These findings suggest that metabolic reprogramming of T cells—and the regulatory networks governing it—may be a more critical determinant of anti-PD-1 response than numerical expansion of specific effector or progenitor exhausted populations.

### 2.2. Metabolic Reprogramming of CD8^+^ T-Cells and Enhanced Macrophage–T-Cell Crosstalk Characterize Anti-PD-1 Responders

**Figure 3:**
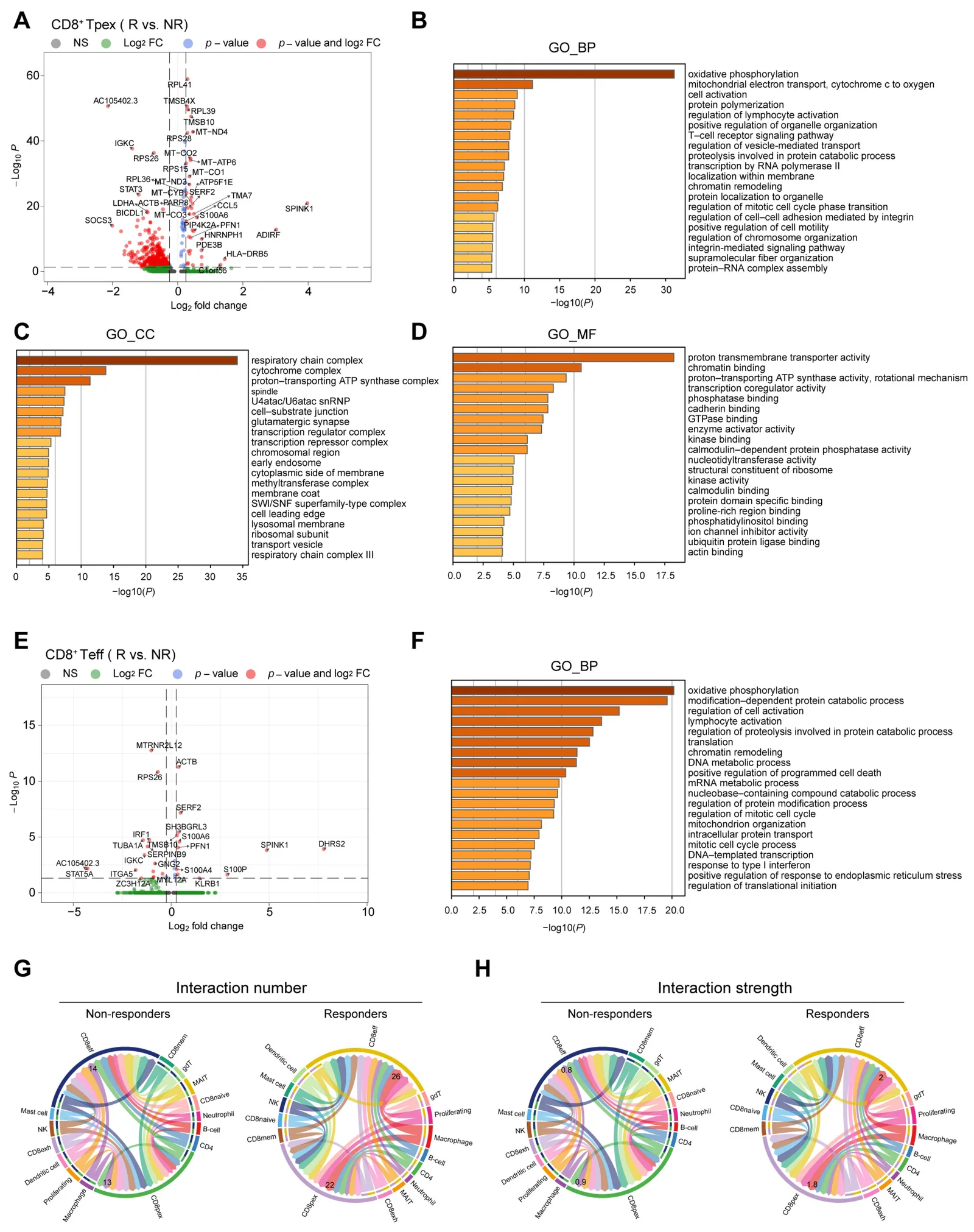
**Metabolic reprogramming of CD8^+^ T-cells and enhanced macrophage–T-cell crosstalk in responders**. (**A**) Volcano plot showing differentially expressed genes in CD8^+^ Tpex cells between R and NR groups. (**B**–**D**) Gene Ontology enrichment analysis of upregulated genes in R-group CD8^+^ Tpex cells, showing enrichment in oxidative phosphorylation, mitochondrial respiration, and ATP generation pathways. BP: Biological Process (**B**), CC: Cellular Component (**C**), MF: Molecular Function (**D**). (**E**) Volcano plot showing differentially expressed genes in CD8^+^ Teff cells between R and NR groups. (**F**) Gene Ontology enrichment analysis of upregulated genes in R-group CD8^+^ Teff cells, showing enrichment in oxidative phosphorylation. (**G**,**H**) Cell–cell communication analysis of immune cells. Macrophage-to-CD8^+^ Tpex and macrophage-to-CD8^+^ Teff interactions were significantly enhanced in the R group.

To identify key regulators of T-cell-mediated anti-PD-1 responses, we performed differential gene expression analysis on CD8^+^ progenitor exhausted T-cells (Tpex), the primary cellular target of PD-1 blockade [22]. Beyond inflammatory mediators like SPINK1, lipogenic genes ADIRF and PDE3B were upregulated in responders, while LDHA, a glycolytic enzyme, was downregulated (Figure 3A). Conversely, non-responder-derived CD8^+^ Tpex exhibited elevated expression of glycolytic and exhaustion-associated genes, consistent with a metabolically suppressed state.

Consistently, Gene Ontology (GO) pathway enrichment analysis further revealed that upregulated genes in responder-derived CD8^+^ Tpex were enriched for oxidative phosphorylation, mitochondrial respiration, and ATP generation pathways (Figure 3B–D). Similarly, in CD8^+^ effector T-cells (Teff), inflammatory and metabolic reprogramming genes—including SPINK1 and DHRS2—were significantly elevated in responders and likewise enriched for oxidative phosphorylation (Figure 3E,F). To identify the cellular source of metabolic support for T-cells, we conducted intercellular communication analysis across immune populations. This revealed significantly enhanced crosstalk between macrophages and both CD8^+^ Tpex and Teff subsets in responders (Figure 3G,H), whereas non-responders displayed markedly attenuated macrophage–T-cell communication, alongside enhanced tumor-cell-mediated MIF signaling as an immunosuppressive alternative. These findings suggest that macrophage–T-cell metabolic interactions underpin the observed T-cell metabolic reprogramming during effective anti-PD-1 therapy, and that the loss of such crosstalk in non-responders may contribute to therapeutic resistance.

### 2.3. M1 Macrophage Polarization Is Driven by CD4^+^ T-Cell-Mediated Antigen Presentation and Metabolic Activation

**Figure 4:**
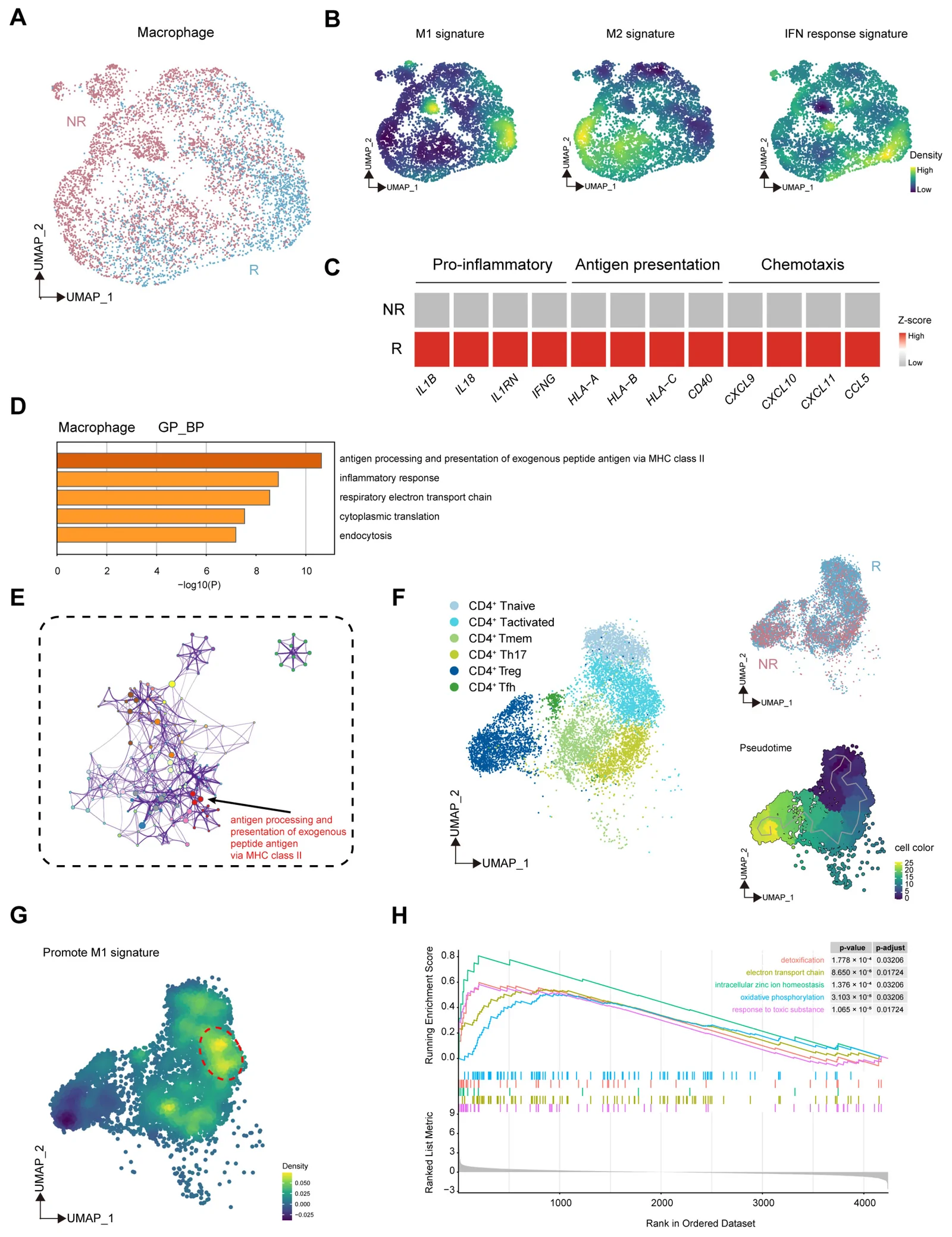
**M1 macrophage polarization is driven by CD4^+^ T-cell activation in responders.** (**A**) UMAP visualization of macrophages in NR and R groups. (**B**) Density plots showing M1 signatures, interferon-stimulated gene signatures, and M2 signatures. M1 and interferon response signatures were enriched in R-group macrophages. M2 signatures predominated in NR-group macrophages. (**C**) Comparison of inflammatory cytokine secretion, antigen presentation, and chemotaxis capacity between R and NR macrophages. (**D**) GO enrichment analysis showing significant enrichment in MHC class II and inflammatory response pathways in R macrophages. (**E**) Network visualization placing MHC class II centrally in enriched pathways. (**F**) Trajectory analysis of CD4^+^ T-cells. (**G**) Signature scoring for Promote M1 signature, demonstrating concentrated signals in the CD4^+^ activated T-cell population enriched in the R group. NR-group CD4^+^ T-cells showed minimal activation signaling. (**H**) GSEA top five pathways for CD4^+^ activated T-cells.

We performed dimensionality reduction and clustering of macrophages and found that M1-associated signatures (CD80, CXCL9, CXCL10, IL1B) and interferon-stimulated gene programs (ISG15, MX1, IFIT1) were enriched in responder-derived macrophages, whereas M2 markers (CD163, MRC1, MSR1) predominated in non-responder macrophages (Figure 4A,B). Differential gene expression analysis revealed significant upregulation of pro-inflammatory, antigen presentation, and chemotaxis pathways in responder macrophages, with pronounced activation of MHC class II-mediated antigen presentation (Figure 4C–E). In contrast, non-responder macrophages exhibited downregulation of these pathways and instead showed enrichment for immunosuppressive and tissue-repair programs.

Given that CD4^+^ T-cells are the principal drivers of MHC-II-dependent macrophage activation, we subjected the expanded CD4^+^ T-cell subsets in responders to dimensionality reduction and pseudotime trajectory analysis. This uncovered a distinct differentiation trajectory from CD4^+^ naïve T-cells toward CD4^+^ activated and CD4^+^ memory states (Figure 4F). We then computed signature scores for IFN-γ (indirect M1 polarization) and CD40L (direct M1 polarization) using IFNG, CD40LG, CD44, and CD69, and found that these signals were concentrated in the CD4^+^ activated T-cell population enriched in responders (Figure 4G), implicating this subset as a key mediator of M1 macrophage polarization. Notably, non-responders lacked this activated CD4^+^ T-cell population and showed minimal IFN-γ and CD40L signaling, correlating with their M2-skewed macrophage phenotype.

Consistent with our earlier findings, Top 5 Gene Set Enrichment Analysis (GSEA) showed that upregulated genes in these CD4^+^ activated T-cells were significantly enriched for electron transport chain and oxidative phosphorylation pathways (Figure 4H), indicating that metabolic activation of CD4^+^ T-cells is coupled to their capacity to license M1 macrophage polarization.

### 2.4. Tumor-Cell-Derived MIF Signaling and SGPL1-Mediated Immunosuppression Shape the Therapeutic Microenvironment

**Figure 5:**
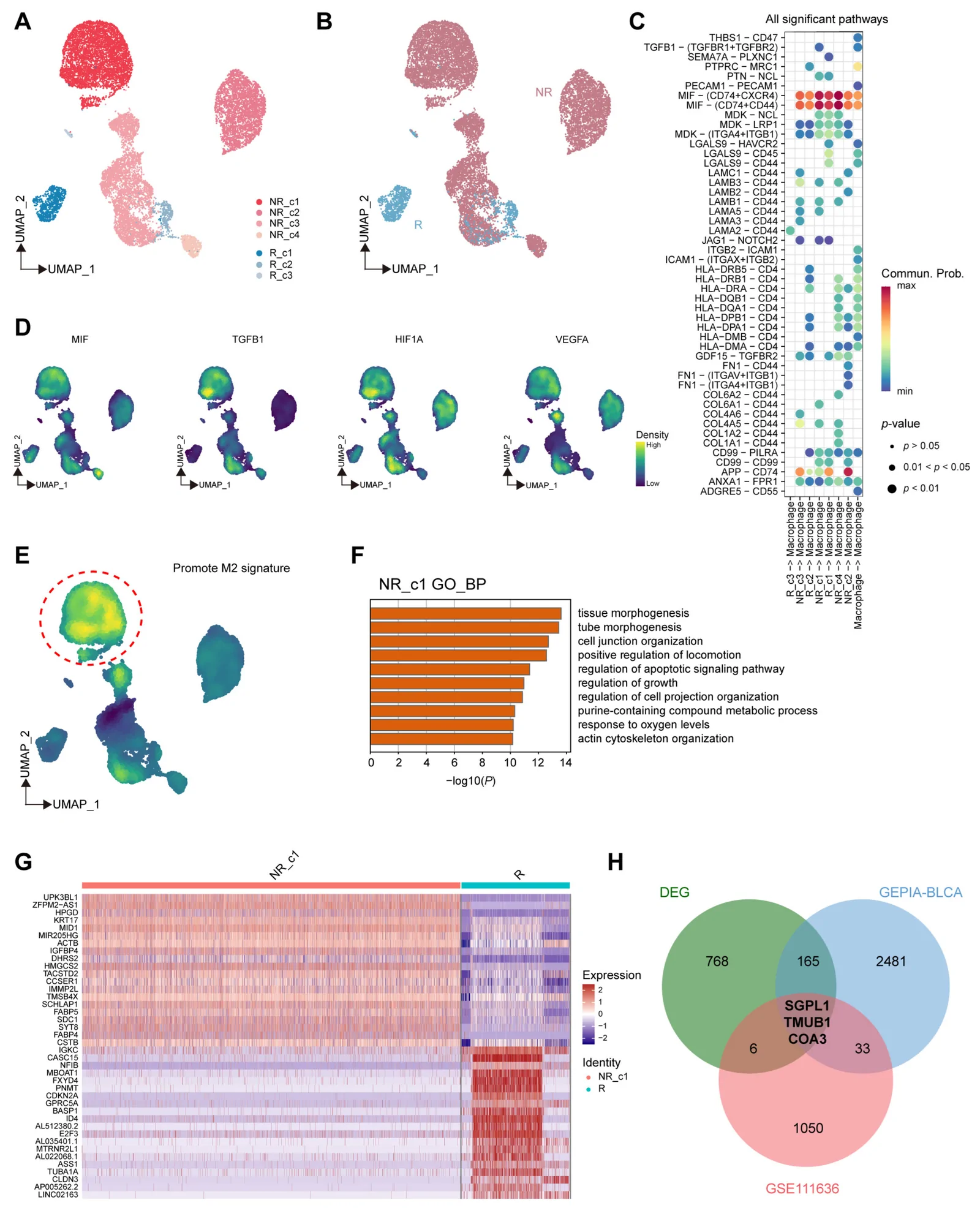
**Tumor cell subclusters and their immunosuppressive communication with macrophages.** (**A**) UMAP visualization of tumor cells after dimensionality reduction and clustering. (**B**) UMAP plots showing the separation of NR and R tumor cell subclusters. (**C**) Cell–cell communication analysis between tumor subclusters and macrophages. The MIF–CD74 pathway was identified as the dominant interaction. (**D**) Feature plots showing the distribution of MIF, TGFB1, HIF1A, and VEGFA signatures across tumor subclusters. MIF was enriched in NR_C1 and NR_C4; TGFB1 in NR_C1; HIF1A in NR_C1–C3; and VEGFA in NR_C2. (**E**) Pro-M2 feature scores across tumor subclusters, showing the highest expression in NR_C1 and low expression in the R group. (**F**) Gene Ontology Biological Process (GO_BP) enrichment analysis of NR_C1 signature genes. (**G**) Heatmap of differentially expressed genes between NR_C1 and R tumor cells. (**H**) Venn diagram showing the intersection of differentially expressed genes from our cohort (|log2FC| > 0.5), GEPIA_BLCA (|log2FC| > 0.5), and GSE111636 (|log2FC| > 0.25), yielding three candidate genes.

To dissect the mechanisms underlying the formation of the M1 macrophage–CD4^+^ T-cell antigen presentation–pro-inflammatory signaling–CD8^+^ Tpex/eff activation axis in responders, we performed dimensionality reduction and clustering of tumor cells. This revealed three tumor cell subclusters in responders and four in non-responders (Figure 5A,B). Intercellular communication analysis between tumor subclusters and macrophages identified MIF–CD74 as the dominant ligand–receptor interaction (Figure 5C). MIF signaling was predominantly enriched in non-responder subclusters NR_C1 and NR_C4, TGFB1 was concentrated in NR_C1, HIF1A in NR_C1–C3, and VEGFA in NR_C2 (Figure 5D). Collectively, pro-M2 features were most pronounced in NR_C1 and markedly attenuated in the responder group (Figure 5E). Gene Ontology Biological Process (GO_BP) enrichment analysis of NR_C1 signature genes revealed enrichment in tissue morphogenesis and cell junction organization pathways (Figure 5F), highlighting NR_C1 as the non-responder subcluster most intimately linked to macrophage polarization. Thus, we conducted differential gene expression analysis between NR_C1 and R groups (Figure 5G). We next intersected our dataset with two publicly available bladder urothelial carcinoma (BLCA) cohorts—GEPIA_BLCA and GSE111636—and identified three significantly altered genes: SGPL1, TMUB1, and COA3 (Figure 5H).

**Figure 6:**
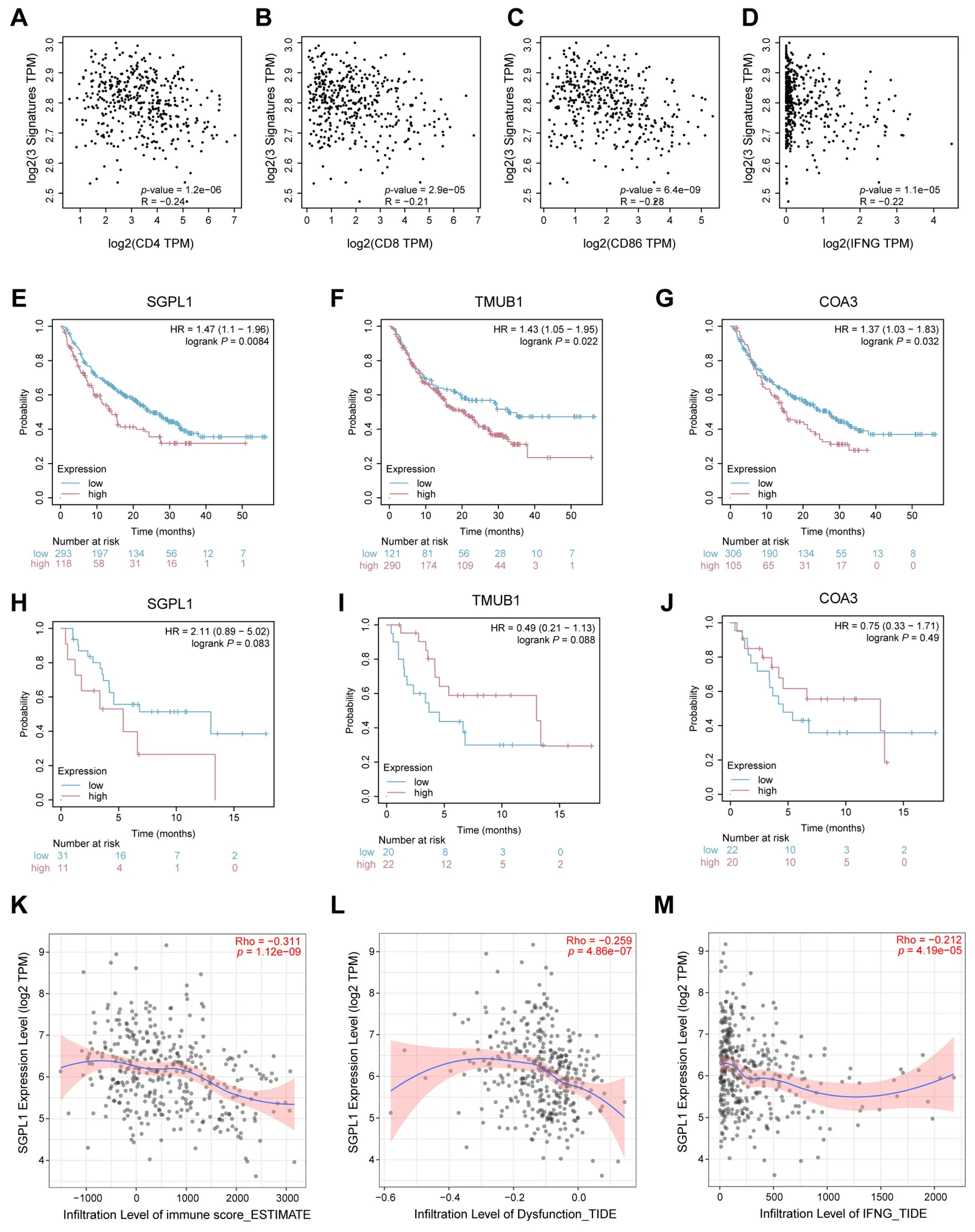
**SGPL1 as a prognostic biomarker and immune suppressor in bladder urothelial carcinoma**. (**A**–**D**) Correlation analysis of the three-gene signature (SGPL1, TMUB1, COA3) with CD4, CD8, CD86, and IFNG expression in BLCA tumors from public databases. All correlations were significantly negative. (**E**–**G**) Kaplan–Meier survival analysis in the KM Plotter pan-cancer immunotherapy cohort. High expression of the SGPL1/TMUB1/COA3was associated with poor prognosis following PD-1 therapy. (**H**–**J**) Kaplan–Meier survival analysis in the KM Plotter BLCA immunotherapy cohort. Only SGPL1 showed a consistent trend, with high expression associated with a higher hazard ratio (HR). (**K**–**M**) Immune analysis in BLCA showing that SGPL1 expression was negatively correlated with immune score, immune dysfunction, and IFNG response.

Correlation analysis of this three-gene signature in BLCA tumors revealed modest negative associations with CD4, CD8, CD86, and IFNG expression (Figure 6A–D). Moreover, high expression of this signature was associated with poor prognosis in the KM Plotter pan-cancer immunotherapy cohort (Figure 6E–G). Notably, in the KM Plotter BLCA immunotherapy cohort, only SGPL1 showed a consistent survival trend: high SGPL1 expression correlated with worse prognosis (Figure 6H–J). Further analysis demonstrated that, within BLCA, SGPL1 expression was significantly negatively correlated with immune score, immune dysfunction, and IFNG response (Figure 6K–M).

### 2.5. SGPL1 Is Associated with Bladder Cancer Progression and Resistance to PD-1 Blockade

**Figure 7:**
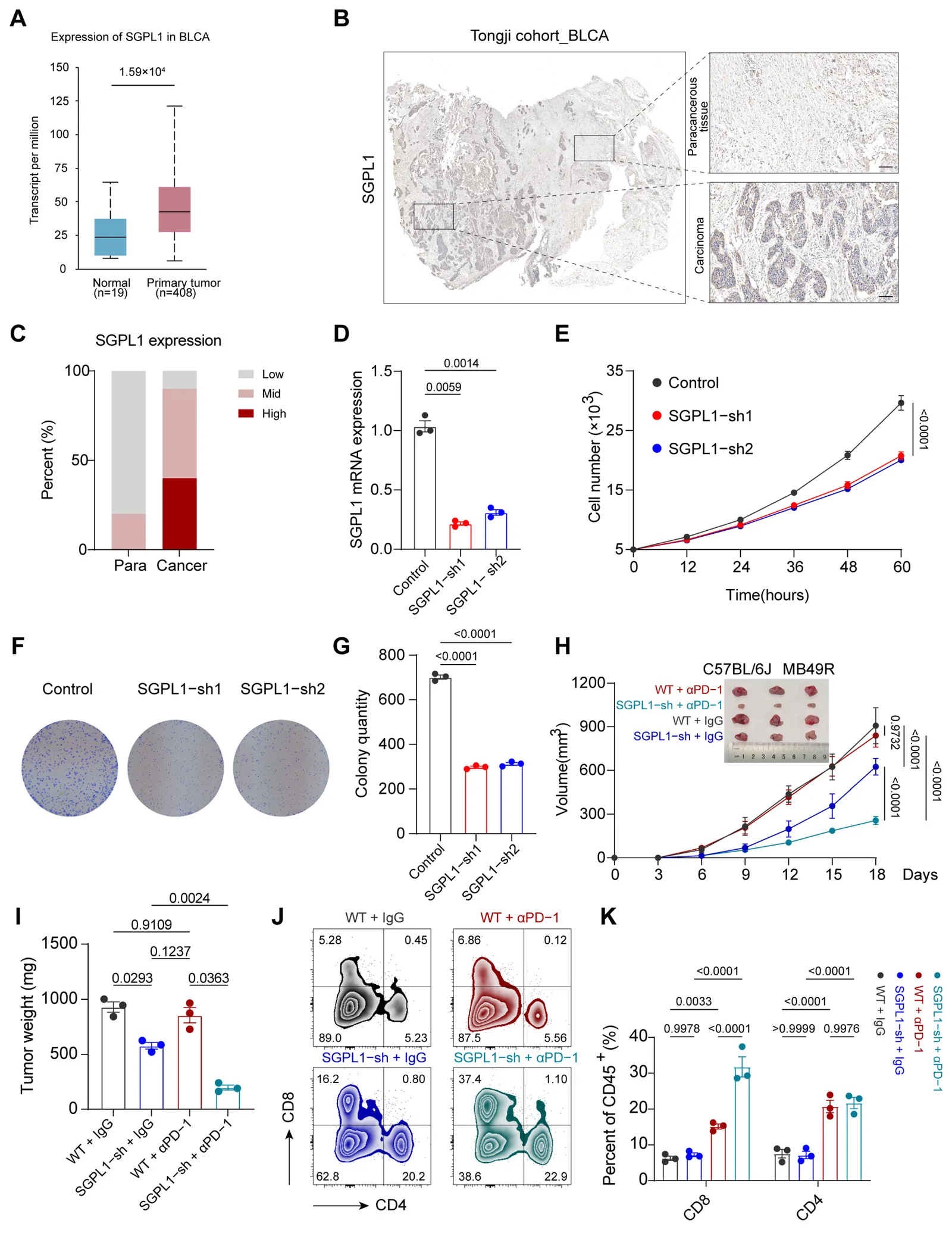
**Functional validation of SGPL1 as a therapeutic target to overcome PD-1 resistance**. (**A**) Analysis of TCGA data showing elevated SGPL1 expression in bladder cancer compared with adjacent normal tissue. (**B**,**C**) Immunohistochemical staining of the Tongji Hospital cohort confirming higher SGPL1 expression in bladder cancer tissue. Scale bar: 100 μm. (**D**) Quantitative PCR validation of SGPL1 knockdown in MB49 cells. (**E**) CCK-8 assay showing that SGPL1 knockdown (SGPL1-sh) inhibited MB49 cell proliferation. (**F**,**G**) Colony formation assay demonstrating reduced clonogenic capacity of SGPL1-sh MB49 cells. (**H**,**I**) In vivo tumor growth curve (**H**) and tumor weight (**I**) in a syngeneic model using MB49-R5 (PD-1-resistant) cells. SGPL1 knockdown significantly restored sensitivity to anti-PD-1 therapy (N = 3). (**J**,**K**) Flow cytometric analysis of tumor-infiltrating lymphocytes (TILs) showing increased proportions of CD4^+^ and CD8^+^ T-cells within the CD45^+^ compartment following SGPL1 knockdown.

To validate the functional role of SGPL1, we first examined its expression profile. Analysis of The Cancer Genome Atlas (TCGA) revealed that SGPL1 was significantly upregulated in bladder cancer compared with adjacent normal tissue (Figure 7A). This finding was corroborated in an independent cohort from Tongji Hospital (Figure 7B,C). We next generated SGPL1-knockdown (SGPL1-KD) MB49 bladder cancer cells and observed a marked reduction in cellular proliferation and colony-forming capacity (Figure 7D–G), indicating that SGPL1 promotes tumor cell intrinsic growth. To model acquired resistance to PD-1 blockade, we derived the fifth-generation PD-1–resistant cell line MB49-R5 from mice subjected to repeated tumor challenge and anti-PD-1 therapy [23]. In vivo tumor experiments demonstrated that SGPL1 knockdown significantly restored sensitivity to PD-1 blockade in MB49-R5 tumors (Figure 7H,I). Flow cytometric analysis further revealed that SGPL1 silencing increased the proportion of CD4^+^ and CD8^+^ T-cells within the CD45^+^ immune compartment (Figure 7J,K). Collectively, these findings establish SGPL1 as a promising metabolic target for combination therapy to overcome resistance to PD-1 blockade in bladder cancer.

## 3. Discussion

Immune checkpoint blockade targeting the PD-1/PD-L1 axis has become a standard treatment for urothelial carcinoma, but 20–30% of patients develop primary or acquired resistance [1,2]. In this study, we used single-cell transcriptomics and functional experiments to investigate the mechanisms underlying resistance. We found that metabolic activation of T-cells, rather than the numerical abundance of specific subsets, was associated with response to anti-PD-1 therapy. In addition, CD4^+^ T-cells were linked to M1 macrophage polarization, and tumor cell-intrinsic SGPL1 was associated with immune evasion and resistance.

Our analysis of T-cell subsets revealed that responders had higher proportions of CD4^+^ activated, memory, and naïve T-cells, whereas CD8^+^ effector and progenitor exhausted cells did not show the expected enrichment. Larger clinical cohorts often correlate therapeutic efficacy with an abundance of specific CD8^+^ subsets [24]. However, our observations suggest that cellular composition alone may not fully determine response in this setting. Instead, the marked upregulation of oxidative phosphorylation and mitochondrial respiration across T-cell subsets in responders highlights the potential importance of metabolic fitness. This aligns with recent studies indicating that metabolic competence is required to sustain T-cell effector function under microenvironmental stress [13,25].

We also found enhanced crosstalk between macrophages and CD8^+^ T-cells in responders. Responder-derived macrophages exhibited M1-associated signatures, while non-responder macrophages showed M2 markers. We traced a potential driver of M1 polarization to CD4^+^ T-cells, which differentiated toward activated and memory states in responders, with IFN-γ and CD40L signals concentrated in the CD4^+^ activated population. This is consistent with the growing appreciation that CD4^+^ T-cells can act as active coordinators of the myeloid microenvironment, rather than playing purely auxiliary helper roles [26,27]. Our cell–cell communication analysis provides a descriptive illustration of this axis in urothelial carcinoma, though the precise mechanistic sequence requires further experimental validation.

To investigate potential tumor-intrinsic factors, we intersected our data with public bladder cancer cohorts and identified SGPL1 as a consistently altered gene associated with poor immunotherapy outcomes. SGPL1 catalyzes the irreversible degradation of sphingosine-1-phosphate (S1P), a bioactive sphingolipid widely characterized for its role in systemic lymphocyte trafficking [28]. While S1P is widely studied for its role in tumor cell proliferation and survival [29], our data suggest it may also influence the tumor immune microenvironment. We speculate that elevated SGPL1 expression in bladder cancer might reduce local S1P availability, potentially affecting immune cell recruitment. Our functional experiments supported this association: SGPL1 knockdown in MB49 cells reduced proliferation, and SGPL1 silencing in a syngeneic-resistant model (MB49-R5) restored sensitivity to anti-PD-1 therapy alongside increased T-cell infiltration. Because we did not directly measure local S1P levels or receptor signaling pathways, these findings represent an initial descriptive link between sphingolipid metabolism and therapeutic outcomes, rather than a definitive causal mechanism.

Our findings have several implications. First, metabolic profiling of tumor-infiltrating T-cells could provide additional parameters for predicting responses to anti-PD-1 therapy. Second, strategies that enhance T-cell metabolic activation or promote M1 macrophage polarization remain interesting areas for future exploration. Third, SGPL1 represents a candidate target for further investigation; its inhibition might offer insights into combination strategies that simultaneously address tumor proliferation and immune infiltration. However, given the limited sample size of our single-cell cohort (*n* = 8) and the descriptive nature of our current findings, these conclusions should be interpreted with caution and require validation in larger, independent cohorts. Several limitations should be noted. First, our single-cell cohort comprised only eight cases, which substantially limits the statistical power and broader generalizability of our findings; these observations need to be prospectively validated in extended clinical cohorts. Second, the precise mechanisms by which SGPL1-mediated pathways affect local T-cell dynamics remain unclear, and the relative contributions of receptor signaling versus direct metabolic effects require further study. Third, while our functional experiments in syngeneic models provide preliminary support for the role of SGPL1, the translational relevance of SGPL1 inhibition as a therapeutic strategy remains speculative at this stage. Nevertheless, our study provides a descriptive framework linking metabolic-immune characteristics in urothelial carcinoma and suggests SGPL1 as one of several candidate targets for future research into overcoming PD-1 resistance.

## 4. Materials and Methods

### 4.1. Study Design and Patient Cohort

This study was approved by the Ethics Committee of Tongji Hospital, Tongji Medical College, Huazhong University of Science and Technology (TJ-202409021). Written informed consent was obtained from all enrolled patients. We collected pre-treatment tumor tissue samples from eight patients with urothelial carcinoma who received pembrolizumab monotherapy at Tongji Hospital.

Response to anti-PD-1 therapy was evaluated according to RECIST v1.1 criteria, with patients classified as responders (R, complete or partial response) or non-responders (NR, stable or progressive disease). The cohort included both primary tumors and lymph node or distant metastases.

### 4.2. Single-Cell RNA Sequencing and Data Preprocessing

Fresh tumor tissues were dissociated into single-cell suspensions using a human tumor dissociation kit (Miltenyi Biotec, Bergisch Gladbach, Germany) according to the manufacturer’s protocol. Single-cell libraries were prepared using the 10x Genomics Chromium Controller and Chromium Single Cell 3′ Reagent Kits v3. Sequencing was performed on an Illumina NovaSeq 6000 platform (Illumina, San Diego, CA, USA). Raw sequencing data were processed using Cell Ranger (10x Genomics, Pleasanton, CA, USA) for demultiplexing, alignment to the GRCh38 human reference genome, and generation of gene expression matrices. Quality control and cell filtering were performed using the Seurat package (v5.5.0) in R. Cells with fewer than 200 detected genes, more than 6000 detected genes, or mitochondrial gene content exceeding 15% were excluded. Doublets were removed using the DoubletFinder package (2.0.6).

Gene expression counts were normalized using the LogNormalize method, and the top 2000 highly variable genes were selected for downstream analysis. Principal component analysis (PCA) was performed, and the first 30 principal components were used for Uniform Manifold Approximation and Projection (UMAP) visualization and clustering. Cell clusters were annotated based on the expression of canonical lineage markers: KRT7 for epithelial tumor cells, PTPRC (CD45) for immune cells, and COL1A1 for stromal cells.

### 4.3. Cell Subclustering and Annotation

Immune cells were extracted and subjected to secondary clustering. T-cells were further stratified into CD4^+^ subsets (activated, memory, naïve), CD8^+^ subsets (effector, progenitor exhausted), and minor populations (γδ T-cells, mucosal-associated invariant T-cells) based on established marker genes. Macrophages were identified and classified according to M1-associated markers (CD80, CXCL9, CXCL10, IL1B), M2-associated markers (CD163, MRC1, MSR1), and interferon-stimulated gene signatures (ISG15, MX1, IFIT1).

### 4.4. Differential Gene Expression and Pathway Enrichment Analysis

Differential gene expression analysis between responders and non-responders was performed using the FindAllMarkers function in Seurat (v5.5.0) with the Wilcoxon rank-sum test. Genes with an adjusted *p*-value < 0.05 and |log2 fold change| > 0.25 were considered significantly differentially expressed. Gene Ontology Biological Process (GO_BP) and Kyoto Encyclopedia of Genes and Genomes (KEGG) pathway enrichment analyses were conducted using the clusterProfiler package. Gene Set Enrichment Analysis (GSEA) was performed using the fgsea package (1.33.1) with gene sets from the Molecular Signatures Database (MSigDB).

### 4.5. Cell–Cell Communication Analysis

Intercellular communication analysis was performed using the CellChat package (1.5.0) in R. Ligand–receptor interactions between cell populations were inferred based on curated databases. The communication probability and strength of signaling pathways were calculated and compared between responder and non-responder groups.

### 4.6. Signature Scoring

Signature scores for IFN-γ, CD40L, stemness, cytotoxicity, and exhaustion were calculated using the AddModuleScore function in Seurat based on established gene sets. The gene sets used were as follows: IFN-γ (IFNG, CD44, CD69), CD40L (CD40LG), stemness (TCF7, LEF1, CCR7), cytotoxicity (LAMP1, GZMK, IFNG, TNF), and exhaustion (TIGIT, LAG3, HAVCR2, PDCD1).

### 4.7. Analysis of Public Datasets

Gene expression data and clinical information for bladder urothelial carcinomas were downloaded from The Cancer Genome Atlas (TCGA-BLCA) and Gene Expression Omnibus (GSE111636). Differential expression analysis between tumor and adjacent normal tissues was performed using the limma package. Survival analysis was conducted using the KM Plotter database for immunotherapy cohorts. Correlation analysis between gene expression and immune markers was performed using the GEPIA2 web server.

### 4.8. Cell Culture and SGPL1 Knockdown

Mouse bladder cancer MB49 cells were cultured in Dulbecco’s Modified Eagle Medium (DMEM) supplemented with 10% fetal bovine serum (FBS) and 1% penicillin–streptomycin at 37 °C with 5% CO_2_. For SGPL1 knockdown, lentiviral vectors expressing short hairpin RNA (shRNA) targeting SGPL1 or a scrambled control sequence were constructed. MB49 cells were infected with lentivirus in the presence of 8 μg/mL polybrene, and stable knockdown cells were selected using 2 μg/mL puromycin. Knockdown efficiency was validated by quantitative real-time PCR (qPCR).

### 4.9. Production and Concentration of Recombinant Lentiviral Particles

Recombinant lentiviral particles were generated through transient co-transfection of HEK293T cells with the transfer vector alongside the second-generation packaging construct psPAX2 and the VSV-G envelope plasmid pMD2.G. The HEK293T cell line was propagated in Dulbecco’s Modified Eagle Medium (DMEM) containing 10% fetal bovine serum and 1% penicillin–streptomycin, and incubated at 37 °C under a humidified atmosphere of 5% CO_2_. Twenty-four hours before transfection, cells were plated onto 10 cm culture dishes at approximately 5 × 10^6^ cells per dish to achieve 70–80% confluency on the day of transfection.

For each 10 cm dish, 10 µg of the transfer plasmid, 7.5 µg of psPAX2, and 2.5 µg of pMD2.G were combined in serum-free Opti-MEM. Linear polyethylenimine was subsequently introduced at a mass ratio of 3:1 relative to the total DNA. Following brief vortexing, the mixture was allowed to stand at ambient temperature for 15–20 min to facilitate complex formation. The resulting DNA–PEI polyplexes were then dispensed onto the cells in a dropwise manner. Six to eight hours later, the transfection mixture was aspirated and replaced with fresh complete growth medium.

Viral-containing supernatants were collected at 48 and 72 h after transfection, combined, and subjected to low-speed centrifugation (3000× *g*, 10 min) to eliminate cellular debris, followed by filtration through a 0.45 µm membrane. The filtrate was further concentrated by ultracentrifugation at 70,000× *g* for 2 h at 4 °C. The resulting viral pellet was gently resuspended in a minimal volume of sterile PBS, divided into aliquots, and preserved at −80 °C until further use.

### 4.10. Cell Proliferation and Colony Formation Assays

Cell proliferation was assessed using the CCK-8 assay. Briefly, 2000 cells per well were seeded onto 96-well plates, and cell viability was measured at 0, 12, 24, 36, 48 and 60 h using the CCK-8 reagent (Dojindo, Kumamoto, Japan) according to the manufacturer’s instructions. Absorbance was measured at 450 nm using a microplate reader. For colony formation assays, 500 cells per well were seeded onto six-well plates and cultured for 10 days. Colonies were fixed with 4% paraformaldehyde, stained with 0.1% crystal violet, and counted using ImageJ software (1.8.0).

### 4.11. Generation of PD-1-Resistant MB49 Cells

PD-1-resistant MB49 cells (MB49-R5) were generated by serial in vivo passaging in C57BL/6 mice. Briefly, MB49 cells were subcutaneously injected into mice, and tumors were treated with anti-PD-1 antibody (200 μg per mouse, twice weekly) upon reaching approximately 100 mm^3^. Tumors that progressed despite treatment were harvested, dissociated, and re-injected into new mice. This process was repeated for five generations to establish the MB49-R5 cell line [23].

### 4.12. In Vivo Tumor Experiments

C57BL/6 mice (6–8 weeks old, male) were purchased from Cyagen Biosciences Inc. (Suzhou, China), and kept at the Animal Center of Tongji Medical College. All protocols for animal experimentation were carried out in adherence to national guidelines and received explicit approval from the Animal Care and Use Committee of Tongji Hospital (protocol identifier TJH-202407024). Animal experiments were designed in accordance with institutional ethical requirements and the principles of Replacement, Reduction, and Refinement (3Rs), using the minimum number of animals necessary for mechanistic evaluation while maintaining statistical validity. Mice were randomly assigned to experimental groups. MB49-R5 cells (5 × 10^6^) with or without SGPL1 knockdown were subcutaneously injected into the right flank. Mice received intraperitoneal injections of anti-PD-1 antibody (BeiGene, Beijing, China, 200 μg per mouse, twice weekly) or isotype control starting on day 7 after tumor inoculation. Tumor size was measured every three days using calipers, and tumor volume was calculated as (length × width^2^)/2. Mice were euthanized when tumors reached 2000 mm^3^ or at the endpoint of the experiment. Tumor weights were recorded at necropsy.

Throughout the in vivo experiments, mice were monitored daily for general health status, including body weight, food and water intake, behavior, activity level, and signs of distress (e.g., lethargy, hunched posture, ruffled fur, or abnormal gait). Body weight was recorded every three days concurrently with tumor measurements. No obvious treatment-related toxicity, significant body weight loss (>20% of initial body weight), or unexpected adverse events were observed in any experimental group throughout the study.

### 4.13. Flow Cytometry

Tumors were harvested, minced, and digested with collagenase IV and DNase I for 45 min at 37 °C. Single-cell suspensions were prepared and stained with fluorochrome-conjugated antibodies against CD45, CD4, and CD8. For each sample, approximately 1 × 10^6^ cells were collected and stained for flow cytometric analysis. Samples were analyzed on a BD FACS Canto II flow cytometer (San Jose, CA, USA), and data were processed using FlowJo software (V10).

### 4.14. Immunohistochemistry

Harvested tumor tissues were immediately fixed in 4% paraformaldehyde (PFA) for 24–48 h at room temperature, followed by dehydration through a graded ethanol series (70%, 80%, 95%, and 100%) and clearing in xylene. Tissues were then embedded in paraffin, and 4 μm thick serial sections were prepared using a rotary microtome. Prior to immunostaining, sections were deparaffinized in xylene and rehydrated through a reverse graded ethanol series (100%, 95%, 80%, 70%) to distilled water. Paraffin-embedded tissue sections were deparaffinized, rehydrated, and subjected to antigen retrieval in citrate buffer. Endogenous peroxidase activity was blocked with 3% hydrogen peroxide. Sections were incubated with anti-SGPL1 primary antibody overnight at 4 °C, followed by horseradish peroxidase-conjugated secondary antibody. Signals were visualized using 3,3′-diaminobenzidine (DAB), and sections were counterstained with hematoxylin.

### 4.15. Quantitative Real-Time PCR

Total RNA was extracted from approximately 5 × 10^5^ cells using TRIzol reagent. To eliminate potential genomic DNA contamination, RNA samples were treated with DNase I (Thermo Fisher Scientific, Waltham, MA, USA) according to the manufacturer’s protocol, followed by purification using the RNeasy Mini Kit (Qiagen, Hilden, Germany). RNA was reverse-transcribed into cDNA using the PrimeScript RT Reagent Kit (Takara Bio, Saint Quentin en Yvelines, France). qPCR was performed using SYBR Green Master Mix on an ABI 7500 Real-Time PCR System. β-actin was selected as the internal reference gene based on its stable expression in our experimental system and its established use as a housekeeping gene in MB49 cell studies. We acknowledge that validation using multiple reference genes could further strengthen normalization accuracy, and we will consider this approach in future studies. The relative expression of SGPL1 was normalized to b-actin using the 2−ΔΔCt method. The primer sequences were as follows: SGPL1 forward, 5′-[TTTCCTCATGGTGTGATGGA]-3′; SGPL1 reverse, 5′-[CCCCAGACAAGCATCCAC]-3′.

### 4.16. Statistical Analysis

Statistical analyses were performed using R (v5.0) and GraphPad Prism (v10.0). Differences between two groups were assessed using unpaired two-tailed Student’s *t*-test or Wilcoxon’s rank-sum test. Correlations were evaluated using Pearson or Spearman correlation analysis. Survival curves were compared using the log-rank test. A *p*-value < 0.05 was considered statistically significant.

## Data Availability

The original contributions presented in the study are included in the article, further inquiries can be directed to the corresponding authors**.** Raw single-cell RNA-seq data have been deposited in the Genome Sequence Archive (GSA-Human: HRA019054), which is publicly accessible at https://ngdc.cncb.ac.cn/gsa-human (accessed on 24 June 2026). To request access, contact the lead contact, Wang Zhihua (zhwang_hust@hotmail.com).

## Abbreviations

The following abbreviations are used in this manuscript:

BLCA	Bladder Urothelial Carcinoma
CCK-8	Cell Counting Kit-8
cDNA	Complementary Deoxyribonucleic Acid
CD40L	CD40 Ligand
COA3	Cytochrome C Oxidase Assembly Factor 3
DAB	3,3′-Diaminobenzidine
GEO	Gene Expression Omnibus
GO	Gene Ontology
GO_BP	Gene Ontology Biological Process
GSEA	Gene Set Enrichment Analysis
HR	Hazard Ratio
IFN-γ	Interferon Gamma
IL1B	Interleukin 1 Beta
ISG	Interferon-Stimulated Gene
KEGG	Kyoto Encyclopedia of Genes and Genomes
MAIT	Mucosal-Associated Invariant T-Cell
MHC	Major Histocompatibility Complex
MIF	Macrophage Migration Inhibitory Factor
MSigDB	Molecular Signatures Database
OXPHOS	Oxidative Phosphorylation
PCA	Principal Component Analysis
PD-1	Programmed Cell Death Protein 1
PD-L1	Programmed Death-Ligand 1
S1P	Sphingosine-1-Phosphate
SGPL1	Sphingosine-1-Phosphate Lyase 1
TCGA	The Cancer Genome Atlas
TGFB1	Transforming Growth Factor Beta 1
Th17	T-Helper 17 Cells
TILs	Tumor-Infiltrating Lymphocytes
TME	Tumor Microenvironment
TMUB1	Transmembrane and Ubiquitin-Like Domain Containing 1
Tpex	Progenitor Exhausted T-Cells
Treg	Regulatory T-Cells
Teff	Effector T-Cells
UC	Urothelial Carcinoma
UMAP	Uniform Manifold Approximation and Projection
VEGFA	Vascular Endothelial Growth Factor A
